# Health survey of adults with hereditary spastic paraparesis compared to population study controls

**DOI:** 10.1186/s13023-016-0469-0

**Published:** 2016-07-13

**Authors:** Krister W. Fjermestad, Øivind J. Kanavin, Eva E. Næss, Lise B. Hoxmark, Grete Hummelvoll

**Affiliations:** Frambu centre for rare disorders, Sandbakkveien 18, 1404 Siggerud, Norway; Department of Psychology, University of Oslo, PO Box 1094 Blindern, 0317 Oslo, Norway

## Abstract

**Background:**

Hereditary spastic paraparesis (HSP) is a rare neurodegenerative condition characterized by slowly progressive spastic weakness of the lower limbs and urinary sphincter dysfunction. Complex HSP involves additional neurologic symptoms and signs like ataxia, extra pyramidal signs, polyneuropathy, and cognitive decline. Little is known about the disease burden for adults with HSP beyond the described core symptoms.

**Methods:**

A cross-sectional survey of 108 adults aged 30 years and older (M_age_ = 57.7 years, SD = 11.5, range 30 to 81; 54.2 % females) recruited from a national center of expertise for rare disorders and a patient advocacy organization in Norway. Self-report data from the HSP sample was compared to self-report data from a large Norwegian population study, HUNT3 (*N* = 46,293), covering health-related variables such as overall life satisfaction, mental wellbeing, memory function, perceived pain, and co-morbid diseases. In addition, the HSP sample reported specific items developed for this study in co-operation with the patient advocacy organization.

**Results:**

The HSP sample more frequently lived alone. Overall, the HSP sample reported lower life satisfaction, lower mental wellbeing and lower social support, as well as poorer memory and sleep, compared to controls. Furthermore, the HSP sample more frequently reported musculoskeletal pain, constipation, and urinary incontinence compared to controls. There was no difference between samples in frequency of physical activity and alcohol and tobacco use. Men with HSP reported higher impact of HSP, lower life satisfaction, and less ability to perform activities of daily living compared to women with HSP.

**Conclusions:**

Adults with HSP experience disease burden on a larger number of areas than previously documented, and men with HSP may represent a particularly vulnerable group.

## Background

The term hereditary spastic paraparesis (HSP) is used to describe a group of neurodegenerative conditions with a high degree of clinical and genetic heterogeneity [1]. HSP can be divided into “pure” and “complex” forms [1, 2]. Pure forms are characterized by slowly progressive spastic weakness in the lower limbs, and urinary sphincter dysfunction. In complex HSP, an additional wide range of neurological features including ataxia, extra pyramidal signs, epilepsy, mental retardation, dementia, and peripheral nerve involvement occurs [1]. In pure HSP, lifespan is not affected [3]. Early disease debut is most often associated with slow progression of the disease, with only a small proportion of affected persons reporting wheelchair use in older age. Late onset, however, tends to imply rapid progression and loss of walking ability from around the age of 60 years and onwards [2, 4, 5].

Several modes of inheritance of HSP have been described, both autosomal dominant, recessive, X linked, or maternal [6]. Pure HSP is mostly inherited in an autosomal dominant manner, whereas complicated forms of HSP tend to be inherited in an autosomal recessive manner [7]. To date, 59 different spastic paraplegia genes are known [8]. Prevalence, based on studies from 16 countries, is estimated to be about 1.8:100 000 [9]. In Norway, where the current survey took place, the estimated combined prevalence of pure and complex HSP has been reported to be 7.4:100 000 [10].

Bladder dysfunction has been described in older adults with HSP [11–13]. A handful of studies have shown disease progression correlates with more severe cognitive impairment [14–17]. The various medical issues that may affect persons with HSP could influence their perceived quality of life (QoL), as well as their psychological and social functioning. Although it is well documented that physical disease, including neurodegenerative disorders, impacts quality of life [18, 19], little is known about the quality of different areas of life for persons affected by HSP. Three studies have investigated QoL and/or psychosocial functioning in adults affected by HSP. In an Estonian study, QoL was found to be significantly lower in 49 persons with HSP aged 20 to 70 years compared to 549 healthy controls, except for the mental health domain [20]. This study did not distinguish between pure and complex forms of HSP. In a German study, physical aspects of health-related QoL were more severely affected than mental aspects in a sample of 143 HSP patients with a mean age of 48 years. However, both spasticity and reduced mobility was associated with lower physical and mental scores. Complex HSP and disease severity predicted lower QoL. Furthermore, both physical and mental scores were lower in patients with complex HSP than in those with pure HSP [21]. In a Brazilian study of 30 HSP patients with the SPG4 gene mutation and a mean age of 48 years, HSP patients reported more depression, pain, and fatigue compared to healthy controls [22].

Beyond these presented findings, there has been limited scientific effort to identify the range of symptoms that may affect persons with HSP. The present study is a broad survey of health and everyday life domains among persons with HSP, including life satisfaction, mental wellbeing, social support, problems with sleep, memory, pain, gastrointestinal/urinary functioning, and ability to perform activities of daily living (ADL). We applied five methodological features to increase interpretability of our findings. First, we recruited a large sample of persons with HSP to increase generalizability to the general HSP population. Second, we recruited our sample through a patient advocacy organization and a resource center for rare disorders, and not from a hospital setting, thereby increasing chances of also examining individuals with fewer symptoms. Third, we developed areas of investigation in co-operation with board members from a HSP patient advocacy organization to target our examination to symptom areas relevant for persons with HSP. Fourth, we applied a number of health-related questions drawn from a large population study, to compare our HSP data to those from a population-based control group. Fifth, we examined potential differences in the HSP sample based on patient age, gender, and pure versus complex HSP.

## Methods

### Sample and procedures

The sample comprised 108 persons with HSP recruited from two sources. The majority (61 %) was recruited from the register of The National Patients’ Association for Hereditary Ataxia and Spastic Paraplegia (NASPA). The remaining sample (39 %) was recruited from the database of Frambu Resource Centre for Rare Disorders (Frambu). Frambu is one of nine state-financed centers of expertise administrated by the Norwegian National Advisory Unit on Rare Disorders. Registration in Frambu’s patient database is voluntary and based on patient consent, and does not require medical referral. Diagnostic confirmation from a medical institution is required to be registered. Questionnaires with prepaid postage were initially sent to the 60 persons with HSP aged 30 years or older who were registered in Frambu’s database. Four weeks later, the questionnaire was sent to 235 NASPA members.

Population data from the third wave of the epidemiological survey “Helseundersøkelsen i Nord-Trøndelag” (HUNT3) was used as control group. HUNT3 is a comprehensive Norwegian survey of health conditions. All inhabitants in Nord-Trøndelag County aged 19 years and above were mailed questionnaires and an invitation to a clinical examination. HUNT3 (2006–2008) comprises the latest population data, providing recent results for gender and age groups comparable to our respondents. The control group comprised 46,293 persons, i.e., all participants aged 30 years and older from the third wave of HUNT3.

The HSP and control samples were not matched beyond age and gender, to examine differences in demographic variables between the HSP sample and controls (e.g., marital and educational status). The study was planned and performed in cooperation with NASPA and approved by The Regional Committee for Medical and Health Research (REK East).

### Measures

The questionnaire comprised three main parts: (a) background and demographic information; (b) health-related questions specific to the HSP diagnosis; and (c) excerpts from HUNT3, covering several themes in relation to mental and physical health and functioning (i.e., life satisfaction, mental wellbeing, pain, sleep, diseases, oral health, gastrointestinal problems, medication use, physical activity, frequency of falling, social support, as well as health service use and satisfaction).

Most themes were covered by a few categorical questions best reported as frequencies rather than as scales. The sleep problems scale had unsatisfactory inter-item reliability, and the items were thus considered separately. We computed combined scale variables with satisfactory inter-item reliability for three themes (Mental wellbeing, α = .79; Memory, α = .88; Gastrointestinal problems, α = .67). Furthermore, we computed a *Total physical impact* variable by adding participants’ scores (*No impact* to *Major impact*) on the 11 symptoms, representing a 12 to 36-range scale from lowest to highest impact.

HSP-specific questions were compiled by the authors on the basis of current medical knowledge on HSP, in co-operation with board members of NASPA. Four board members of NASPA provided feedback on a preliminary version of the questionnaire. This feedback influenced selection of both HSP-specific questions and HUNT3 variables. In co-operation with NASPA we decided not to include questions on typical symptoms of complex HSP, to reduce the burden on participants. Controls were not presented with the HSP-specific questions.

### Data analysis

Data were analyzed using IBM SPSS version 21.0. Comparisons between the HSP sample and controls were done by calculating Pearson’s chi-square to estimate whether frequencies were significantly different between the samples. To reduce expected frequencies below 5, some response categories were combined for some variables (e.g., *often* and *very often*) when required. When relevant, effect size differences were calculated as M_HSP sample_ - M_controls_/pooled standard deviation. We used the following cut off values to interpret magnitude of effect sizes: *d* = 0.2 (small), *d* = 0.5 (medium), and *d* = 0.8 (large) [23]. Effect sizes were only calculated if differences were significant at or below the *p* = .05-level.

## Results

The response rate from patients in the Frambu database was 80 %. The exact response rate from the NASPA register is not known, as NASPA also has members with hereditary ataxias, who were asked to ignore the request. The response rate in HUNT3 was 54 %.

### Comparison of samples

The mean age between the HSP sample and controls was comparable (*M*_HSP_ = 57.7 years, *SD* = 11.5 vs. *M*_controls_ = 55.9 years, *SD* = 14.0, *d* = 0.13). The gender distribution between the HSP sample and controls was not significantly different (*female ratio*_HSP_ = 53.7 % vs. *female ratio*_controls_ = 54.2 %; (*χ*^*2*^ (1) = 0.011, *p* = .916).

Within the HSP sample, participants recruited from NASPA were significantly older than participants recruited from Frambu (*M*_NASPA_ = 59.7 years, *SD* = 11.5 vs. *M*_Frambu_ = 54.6 years, *SD* = 10.9, *d* = 0.46). The gender distribution was not significantly different dependent on recruitment setting (*female ratio*_NASPA_ = 50.8 % vs. *female ratio*_Frambu_ = 59.5 %; (*χ*^*2*^ (1) = 0.788, *p* = .354).

### Background information

See Table 1 for information on marital status, educational level, and main source of income for the HSP sample. Within the HSP sample, women more frequently reported to be married or cohabiting compared to men (*χ*^*2*^ (1) = 4.585, *p* < .05). Apart from this, there was no gender difference in the HSP sample in terms of background variables. In the HSP sample, 15.7 % reported to live with children.

**Table 1:** Background variables for the HSP sample

	Total	Men	Women
	(*N* = 107^a^)	(*N* = 49)	(*N* = 58)
Marital status			
Single	23.1 %	30.6 %	17.2 %
Married/cohabiting	54.9 %	44.9 %	65.5 %^b^
Divorced	15.7 %	18.4 %	13.8 %
Widowed	4.6 %	6.1 %	3.4 %
Education level			
Primary school	11.1 %	6.1 %	15.8 %
Secondary school	34.3 %	34.7 %	33.3 %
High school	23.1 %	22.4 %	24.6 %
<3years University degree	15.7 %	16.3 %	15.8 %
>3years University degree	13.9 %	20.4 %	8.8 %
Other	0.9 %	0 %	1.8 %
Main source of income			
Paid salary	18.5 %	14.3 %	22.4 %
Disability pension	53.7 %	51.0 %	55.2 %
Retirement pension	24.1 %	30.6 %	19.0 %
Sickness benefits	2.8 %	2.0 %	3.4 %
Body mass index (BMI)	M = 25.5 (SD = 4.5)	M = 26.2 (SD = 4.0)	M = 24.9 (SD = 4.8)

*Note*. ^a^Bacground information was missing for one participant. ^b^Gender difference was significant at the .05-level (Pearson’s chi-square)

### HSP compared to controls

Comparing the HSP sample to controls, there were significant differences in family status (*χ*^*2*^ (3) = 1711.46, *p* < .001). Participants in the HSP sample less frequently lived with a partner/spouse (*χ*^*2*^ (1) = 5.37, *p* < .05), and more frequently lived alone (*χ*^*2*^ (1) = 32.07, *p* < .001). There was no difference between the samples in frequency of living with parents (*χ*^*2*^ (1) = 0.21, *p* = .647) or children (*χ*^*2*^ (1) = 0.51, *p* = .477).

Overall, the HSP sample reported lower life satisfaction, lower mental wellbeing and lower social support, as well as poorer memory and sleep, compared to controls. Furthermore, the HSP sample more frequently reported musculoskeletal pain, constipation, and urinary incontinence compared to controls. There was no difference between samples in frequency of physical activity and alcohol and tobacco use. A detailed summary of the main findings regarding the HSP sample compared to controls is presented in Table 2.

**Table 2:** HSP sample compared to controls: Overview of main results

Main finding HSP sample	HSP sample compared to controls
Overall life satisfaction	
M = 3.6 (*SD* = 1.2, *range* 1 to 7)^a^	HSP sample poorer (*χ* ^*2*^ (1) = 210.20, *p* < .001).
Mental wellbeing	
M = 11.4 (*SD* = 3.3, range 6 to 21)	HSP scored lower than controls on 5 of 6 items (*χ* ^*2*^ (1) range 13.38 to 65.95, all *p* < .001).
Memory	
M = 15.2 (SD = 4.0, range 9 to 27)	HSP more frequently reported problems on 6 of 9 items (*χ* ^*2*^ (1) range 4.21 to 26.306, all *p* < .05)
Sleep	
Percentage of participants who rated *several times a week*: daytime drowsiness: 35 %; frequent night awakenings 36 %; trouble falling asleep 28 %; waking up early 19 %.	HSP reported more sleep problems on all items (*χ* ^*2*^ (1) range 6.39 to 59.26, all *p* < .05).
Pain	
70 participants (64.8 %) confirmed chronic pain of >3 months duration in the past year. Most frequent pain sites: feet, knees, lower back, and hips.	HSP confirmed more frequent musculoskeletal pain (*χ* ^*2*^ (1) = 6.51, *p* < .05). HSP confirmed more frequent pain in the lower body pain sites (*χ* ^*2*^ (1) range 5.067 to 61.636, all *p* < .05). Controls more frequent pain in the upper body pain sites (*χ* ^*2*^ (1) range 7.817 to 26.141, all *p* < .05).
Comorbid disease prevalence	
Most frequently reported diseases were mental health problems 19 %; osteoarthritis 14 %; hand eczema 11 %; psoriasis 10 %; asthma 8 %; and brain hemorrhage 7 %.	HSP more frequently reported brain hemorrhage (*χ* ^*2*^ (1) = 5.284, *p* < .05) and psoriasis (*χ* ^*2*^ (1) = 4.324, *p* < .05).
Gastrointestinal problems	
Percentage of participants who reported *much problems*: constipation 14 %; alternating constipation and diarrhea 7 %; bloating 7 %; heartburn 6 %; diarrhea; and nausea 0 %. 11 % confirmed fecal incontinence weekly or daily.	HSP more frequently reported *much problems* on alternating constipation and diarrhea (*χ* ^*2*^ (1) = 9.163, *p* < .05) and constipation (*χ* ^*2*^ (1) = 11.032, *p* < .05). HSP more frequently reported fecal incontinence (*χ* ^*2*^ (1) = 26.253, *p* < .001).
Urinary problems	
52 % confirmed urinary incontinence	HSP more frequently reported urinary incontinence (*χ* ^*2*^ (1) = 5.427, *p* < .05)
Oral health	
67 % rated their oral health as *good* or *very good*, while 13 % rated their oral health as *poor* or *very poor*.	No difference, but HSP reported more frequent dental visits during the last year compared to controls (*χ* ^*2*^ (1) = 4.466, *p* < .05).
Physical activity	
Reported frequency of physical activity: daily 18 %; 2–3 times pr. week 33 %; once a week 22 %; less than once a week 14 %; and never 11 %.^b^	No difference in frequency. HSP spent more hours sitting daily compared to controls (M_HSP_ = 9.0 vs. M_controls_ = 5.7, effect size difference *d* = 1.18).
Medication use	
Percentage of participants who reported taking nonprescription medicines 1–3 times weekly: for general pain 31 %; for constipation 17 %; for headache 16 %; and for heartburn 10 %.^c^	HSP more frequently reported taking medication for constipation (*χ* ^*2*^ (1) = 30.685, *p* < .001) and general pain (*χ* ^*2*^ (1) = 4.068, *p* < .05). Otherwise no difference.
Alcohol and tobacco use	
Percentage of participants who reported drinking alcohol at least 2–3 times pr. week 20 %, never drinking alcohol 11 %, smoking daily 11 %.	No difference.
Social support	
Percentage of participants who confirmed practical support 82 % and emotional support 80 %^d^	HSP reported lower practical support (*χ* ^*2*^ (1) = 36.07, *p* < .001) and lower emotional support (*χ* ^*2*^ (1) = 13.59, *p* < .001).

*Note. M* mean, *SD* standard deviation. No age or gender difference unless indicated. ^a^ = males reported higher impact than females. ^b^ = older participants spent more hours sitting daily. ^c^ = older participants took more laxatives. ^d^ = older participants reported lower emotional support

### HSP-specific results

The majority (58 %) reported that HSP was genetically confirmed. A quarter (24 %) reported that this was not the case, while 16 % reported they did not know. The majority (66 %) reported that one or more family members also had HSP. With multiple categories possible, the most frequently reported family members with HSP were siblings (*n* = 35), mothers (*n* = 25), children (*n* = 22), and fathers (*n* = 19).

#### Overall impact of HSP

Participants rated the overall impact of having HSP on a scale from 0 to 10, with 10 indicating the most negative impact. The mean rating was 7.2 (*SD* = 2.2), with males (m) rating a higher overall negative impact than females (f) (*M*_m_ = 7.8, *SD*_m_ = 2.1; *M*_f_ = 6.7, *SD*_f_ = 2.3; *t* = 2.399, *p* < .05; *d* = 0.50). The correlation between age and HSP impact was non-significant (*r* = .15, *ns*).

#### Physical impact

**Fig. 1:**
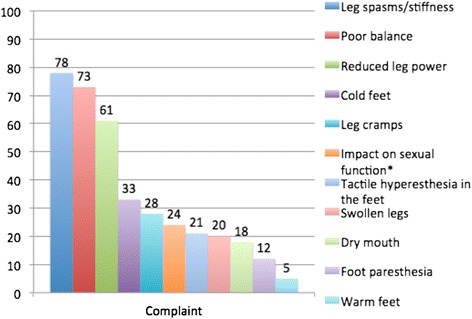
Percentage of 108 persons with HSP reporting “major impact” of physical complaints. *Note*. *Significant gender difference (*p* < .001)

Figure 1 shows the percentages of participants who rated “major impact” of 11 physical symptoms. There was no gender difference except for the frequency of reporting impact on sexual function, which was more often reported by males with HSP compared to females with HSP (*χ*^*2*^ (1) = 10.299, *p* < .001).

The mean score for all participants on the 12–36 range physical impact scale was 24.0 (*SD* = 4.8). There was no significant gender difference (*M*_m_ = 24.7, *SD*_m_ = 5.2; *M*_f_ = 23.4, *SD*_f_ = 4.4; *t* = 1.447, *ns*). Forty-two women responded to whether physical symptoms got worse with menstruation (including in retrospect for those beyond menstrual age). Of these 42 women, eight confirmed a low degree of worsening, three confirmed high degree of worsening, while 31 rated no worsening.

#### Mobility and activities of daily living

In terms of mobility, 35 % reported to walk without aids outdoors, while 56 % reported to walk without aids indoors. Around a third (31 %) reported to use a wheelchair indoors, while 45 % reported to use a wheelchair outdoors. The majority (80 %) confirmed having a driver’s license. See Table 3 for overview of the percentage of participants who reported incapability to conduct activities of daily living (ADL) without assistance.

**Table 3:** Ability to perform activities of daily living among 108 persons with HSP

Activity	Percentage who confirmed not being able to perform activity without assistance		
	Total sample	Pure HSP	Complex HSP
	*N* = 108	*N* = 75	*N* = 32
Walking indoors, ground floor	14 %	12 %	16 %
Visit the toilet	6 %	4 %	6 %
Body washing	5 %	1 %	9 %
Showering/bathing	8 %	4 %	16 %^a^
Dress-undress	5 %	3 %	6 %
Get out of bed/get into bed	5 %	3 %	6 %
Eating	2 %	1 %	0 %
Preparing warm meals	15 %	8 %	28 %^a^
Easy household chores	13 %^b^	8 %	22 %^a^
Complex household chores	46 %	37 %	66 %^a^
Do the laundry	19 %^b^	11 %	34 %^a^
Grocery shopping	21 %	13 %	38 %^a^
Paying bills	12 %	7 %	22 %^a^
Taking medications	5 %	3 %	6 %
Getting outside daily	13 %	9 %	19 %
Taking the bus	51 %	41 %	72 %^a^
Driving	12 %	9 %	19 %^a^

Note. ^a^Participants with complex HSP significantly more frequently confirmed not being able to perform activity unassisted (all *χ* ^*2*^ (1) >3.935, all *p* < .05). ^b^Men significantly more frequently confirmed not being able to perform activity unassisted (all *p* < .05)

We computed a total activities of daily living (ADL) score by adding the number of activities participants reported not being able to perform. The mean score was 2.7 activities, (*SD* = 3.6, *range* 0 *to* 16). Men reported more activities they could not perform without assistance compared to women (*M*_m_ = 3.5, *SD*_m_ = 4.3; *M*_f_ = 2.0, *SD*_f_ = 2.9; *t* = 2.101, *p* < .05; *d* = 0.16). Fisher’s exact tests showed that the two activities men reported to be able to perform less frequently compared to women were simple household chores (*p* < .05) and laundry (*p* < .001).

#### Frequency of falling

In the HSP sample, 47 % reported to have fallen in the last 3 months. There was no gender difference in frequency of falling (*χ*^*2*^ (1) = 1.375, *ns*). There were no significant age difference between those who confirmed having fallen and those who did not (*t* = 0.588, *ns*).

#### HSP sample medication use

In the HSP sample, 15 % reported using Botox injections, 10 % reported using a baclofen pump, and 33 % reported taking oral spasmolytics. Of these, the percentages of participants reporting having some or large effect of the medication were 83 % for Botox, 86 % for baclofen pump, and 82 % for oral spasmolytics.

### Gender differences within the HSP sample

There were no significant gender differences on any of the variables shown in Table 2, except overall life satisfaction. Males rated significantly lower life satisfaction compared to females (*M*_m_ = 3.9, *SD* = 1.1, *M*_f_ = 3.3, *SD*_f_ = 1.3; *t* = −2.157, *p* < .05; *d* = 0.40).

### Pure versus complex HSP

The majority of the HSP sample (69 %) reported to be “mainly affected in the lower body”, which was used a proxy for pure HSP. We examined whether there were significant differences based on this distinction, and found that this was the case for three variables. Participants with proxy complex HSP reported a higher overall impact of HSP (*M*_pure_ = 6.9, *SD*_pure_ = 2.1 vs. *M*_complex_ = 8.1, *SD*_complex_ = 2.3; *t* = 2.669, *p* <. 05; *d* = 0.55). Furthermore, those with proxy complex HSP were diagnosed at a later age compared to those with proxy pure HSP (*M*_pure_ = 40.4 years, *SD*_pure_ = 15.8 vs. *M*_complex_ = 48.7 years, *SD*_complex_ = 15.4; *t* = 2.435, *p* <. 05; *d* = 0.51). Finally, those with proxy complex HSP reported more ADL that they were not able to perform without assistance (*M*_pure_ = 1.9 activities, *SD*_pure_ = 2.9 vs. *M*_complex_ = 4.4 activities, *SD*_complex_ = 4.0; *t* = 3.439, *p* <. 001; *d* = 0.74). Significantly fewer participants with proxy complex HSP reported to have a driver license compared to those with proxy pure HSP (*χ*^*2*^ (1) = 15.867, *p* < .001). See Table 3 for differences between the pure and complex types in terms of ability to perform activities of daily living.

There was no difference between the proxy pure/complex types in terms of age, total body impact, pain, mental well-being, memory, gastrointestinal/urinary problems, number of additional diseases, BMI, or physical activity.

### Correlations between variables in the HSP sample

See Table 4 for correlations between the scale variables for the HSP sample. Note that apart from age of onset, age was only significantly correlated with physical impact, and no other health variable. In fact, physical impact was the factor with most significant correlations with other health variables.

**Table 4:** Correlations between variables among 108 adults with HSP

	1.	2.	3.	4.	5.	6.	7.	8.	9.	10.	11.	12.	13.
1. Age	1												
2. Age of onset	.60**	1											
3. Education	-.16	-.22*	1										
4. BMI	-.19	-.20*	.11	1									
5. Overall HSP impact^a^	.15	.08	.03	-.04	1								
6. Physical impact^a^	.25*	.04	-.07	.02	.37**	1							
7. Mental wellbeing	.03	-.01	-.22*	-.14	.22*	.34**	1						
8. Life satisfaction^a^	.03	.05	-.23*	-.12	.54**	.39**	.57**	1					
9. Physical activity	-.06	.07	.14	-.07	-.08	-.15	-.14	-.06	1				
10. Memory problems^a^	.13	.15	-.16	.04	.13	.28**	.30**	.19	-.03	1			
11. Gastrointestinal problems^a^	-.02	-.02	-.07	-.09	.08	.36**	.16	.25*	-.07	.33**	1		
12. Number of diseases	.13	.01	-.11	.05	.09	.23*	-.01	-.01	-.27**	.11	.16	1	
13. Pain^a^	-.05	-.00	-.00	.02	.26*	.48**	.22	.27*	-.12	.22*	.29*	.18	1
14. Activities of daily living^a^	.40**	.21	-.06	-.22	.23	.21	.14	.18	-.10	.29*	.18	.23	.29*

*Note*. ^a^Higher scores indicate more problems. *Correlation is significant at the *p* < .05-level. **Correlation is significant at the *p* < .001 level

## Discussion

The present report is a comprehensive survey of multiple health variables in a large sample of adults aged 30 years and older with HSP. Several variables were drawn from HUNT3, a large population survey, allowing comparison of results from the HSP sample to population controls. Compared to controls, persons with HSP reported lower scores on life satisfaction, mental wellbeing, as well as perceived practical and emotional support. Furthermore, compared to controls, persons with HSP reported more problems with memory, sleep, gastrointestinal and urinary function, and pain in the lower body. Our results showed persons with HSP experience large total physical impact of their disorder. This total impact was significantly correlated with age, mental wellbeing, memory problems, gastrointestinal problems, extent of pain, number of co-morbid diseases, and life satisfaction. Thus, the disease burden for adults with HSP is multifaceted, and involves problem areas not previously documented.

Gastrointestinal problems for persons with HSP have previously been documented in a Dutch study, in which combined fecal and urinary incontinence was identified among seven of 18 patients aged 13 to 46 years with complex HSP, specifically SPG11 mutation [24]. In our HSP sample, gastrointestinal problems were significantly correlated with perceived quality of life. This in line with findings from a review of adult patients with central neurological disorders like Parkinson’s disease, Alzheimer’s disease and multiple sclerosis, in which impaired bowel function was shown to increase anxiety and reduce quality of life in [25]. The identified gastrointestinal problems for persons with HSP warrant further research and clinical attention.

Our results also showed considerable impact on activities of daily living for persons with HSP. Over half the sample reported not being able to take the bus, and nearly half the sample reported not being able to do more than basic house chores. We were surprised to find that age was only correlated with total physical impact and ability to perform ADL, and not with any other health-related variable. This implies that the burden of disease experienced by adults with HSP is considerable across the lifespan. Importantly, older participants reported less practical support compared to younger participants, possibly indicating a particular need for the older HSP group.

We uncovered some important gender differences within the HSP sample. Specifically, men reported significantly higher overall impact of HSP, higher impact on sexual function, more ADL they could not perform without assistance, and lower overall life satisfaction compared to women with HSP. Possibly, the features of HSP are in larger conflict with typical gender expectations for men compared to women, as the stereotypical and traditional Western male gender role ideologies tend to favor physical capabilities as well as personal autonomy [26]. On the other hand, we did not find gender differences that could be expected based on previous findings, such as in terms of perceived social support, which has been shown to be lower among males [27]. In sum, our results indicate that men with HSP represent a particularly vulnerable group in terms of overall HSP impact and quality of life.

The current study has some limitations. First, we rely on self-reported diagnosis and not genetic confirmation. Although we have no reason to believe non-HSP patients responded, we cannot rule out this possibility. Second, beyond self-reported memory problems we did not assess cognitive abilities among participants. Cognitive deficits are documented among HSP patients and may impact both symptoms and self-report accuracy. Third, relying on self-report excluded possibilities of cross-referencing and validating data with medical records. Fourth, we used “only lower body affect” as a proxy for pure HSP. In a questionnaire-based study, this was perceived to be the most reliable distinction between pure and complex. The prevalence of the two forms in our study is in accordance with a previous survey [21]. However, the validity of this distinction is uncertain, so our findings regarding differences between pure and complex HSP should be interpreted with caution. Finally, it is likely that some persons in the control group experience neuromotor problems, but due to the substantial size of the control group this is unlikely to have influenced results.

In spite of these limitations, the considerable sample size, the inclusion of a large population-based control group, and the involvement of a patient advocacy organization in the development of measures, indicate the results of the present report represent important and relevant insights into self-perceived functioning in patients with HSP. Future studies should examine the perspective of the providers of health services to patients with HSP, to identify potential knowledge gaps about this patient population.

## Conclusion

The main clinical implications of the present report are that HSP patients should be assessed on a wide range of areas of functioning. Men with HSP and persons with complex HSP may be in particular need of such intervention. Mental health was significantly correlated with physical impact and overall impact of HSP. Although our design prevents us from examining causality, health professionals should also assess mental health in patients with HSP. Future studies should examine longitudinal development of the problem areas identified for patients with HSP, to provide better standards for care.

## Abbreviations

ADL, activities of daily living; HSP, hereditary spastic paraparesis; HUNT3, helseundersøkelsen i Nord-Trøndelag (Health Survey of Nord-Trøndelag); M, mean; NASPA, the national patients’ association for hereditary ataxia and spastic paraplegia; QoL, quality of life; SD, standard deviation
